# Exposure to West Nile Virus Increases Bacterial Diversity and Immune Gene Expression in *Culex pipiens*

**DOI:** 10.3390/v7102886

**Published:** 2015-10-27

**Authors:** Steven D. Zink, Greta A. Van Slyke, Michael J. Palumbo, Laura D. Kramer, Alexander T. Ciota

**Affiliations:** 1 Griffin Laboratory, Wadsworth Center, New York State Department of Health, Slingerlands, NY 12159, USA; steven.zink@health.ny.gov (S.D.Z.); greta.vanslyke@health.ny.gov (G.A.V.S.); laura.kramer@health.ny.gov (L.D.K.); 2 Wadsworth Center Bioinformatics Core, Wadsworth Center, New York State Department of Health, Albany, NY 12222, USA; michael.palumbo@health.ny.gov

**Keywords:** West Nile virus, arbovirus, microbiome, *Culex* mosquitoes, invertebrate immunity

## Abstract

Complex interactions between microbial residents of mosquitoes and arboviruses are likely to influence many aspects of vectorial capacity and could potentially have profound effects on patterns of arbovirus transmission. Such interactions have not been well studied for *West Nile virus* (WNV; *Flaviviridae*, *Flavivirus*) and *Culex* spp. mosquitoes. We utilized next-generation sequencing of 16S ribosomal RNA bacterial genes derived from *Culex pipiens Linnaeus* following WNV exposure and/or infection and compared bacterial populations and broad immune responses to unexposed mosquitoes. Our results demonstrate that WNV infection increases the diversity of bacterial populations and is associated with up-regulation of classical invertebrate immune pathways including RNA interference (RNAi), Toll, and Jak-STAT (Janus kinase-Signal Transducer and Activator of Transcription). In addition, WNV exposure alone, without the establishment of infection, results in similar alterations to microbial and immune signatures, although to a lesser extent. Multiple bacterial genera were found in greater abundance in WNV-exposed and/or infected mosquitoes, yet the most consistent and notable was the genus *Serratia*.

## 1. Introduction

*West Nile virus* (WNV; *Flaviviridae*, *Flavivirus*) is the most prevalent arthropod-borne virus (arbovirus) in the U.S. and the most geographically widespread arbovirus in the world. Although the majority of WNV infections go undiagnosed, over 40,000 cases of disease and 1400 deaths have been attributed to WNV over the last 15 years in the U.S. WNV is maintained in nature in an enzootic cycle between *Culex* spp. mosquitoes and birds. *Culex pipiens Linnaeus* is the primary enzootic vector of WNV in the northeastern U.S. and is likely to also play a principal role in both human spillover and seasonal maintenance (reviewed in [1,2]).

Mosquitoes are constantly exposed to a diverse range of microbes which directly or indirectly interact and can significantly alter innate immunity and fitness [3,4,5,6]. Larval habitats are home to diverse microbial communities and although many bacterial species may be found exclusively in mosquito larvae, numerous species are also maintained transstadially [7,8,9,10,11]. Adult mosquitoes may be exposed to additional bacteria through sugar feeding and breaks in their cuticle. While the essential function of the nutrient rich blood meal is to facilitate egg development, it also results in substantial alterations to microbial diversity and load [12]. These microbial alterations are, therefore, inherently bound to arbovirus exposure and infection.

Pioneered largely by studies of *Drosophila*, our understanding of the complexity and redundancy of the multifunctional invertebrate immune system has greatly increased in recent years [13]. Although RNA interference (RNAi) may be the primary immune response to arboviruses in mosquitoes [14], classic innate immune responses, including the Toll, Imd (Immune Deficiency), and Jak-STAT(Janus kinase-Signal Transducer and Activator of Transcription) signaling pathways, have also been implicated in control of viral infections of invertebrates, including mosquitoes [15]. In addition, the historic assumption of a benign relationship between arboviruses and their vectors has been challenged, with documented associations between arbovirus exposure and alterations to mosquito life-history traits, including effects of WNV exposure on longevity, blood feeding, and fecundity [16,17,18]. Understanding the complex interactions between microbial communities, arboviruses, and mosquito immunity has direct implications in our understanding of the factors contributing to vectorial capacity, including arbovirus competence, mosquito survival, and blood feeding behavior. In addition, as demonstrated with the introduction of *Wolbachia*-infected *Aedes aegypti* to limit *Dengue virus* (DENV) transmission [17,19,20,21], characterizing these relationships can ultimately result in novel control strategies.

Although the microbiome of the midgut has been shown to influence the fitness of arboviruses within the mosquito [3,22], the specifics of these interactions remain largely uncharacterized, particularly in the *Culex*-WNV system. In addition, past studies have focused primarily on assessing the influence of the bacterial community on arbovirus competence, yet have largely ignored the effect of the virus on microbial composition. In this study we utilized next-generation sequencing of 16S bacterial genes derived from *Cx. pipiens* following WNV exposure and/or infection and compared bacterial populations and broad immune responses to unexposed mosquitoes. These data demonstrate that unique microbial signatures are associated with WNV exposure and infection, providing insight into the multifaceted interactions between arboviruses, microbial communities, and mosquito immunity which have broad implications for our understanding of the complexity of transmission of WNV and other arboviruses.

## 2. Materials and Methods

### 2.1. Mosquito Blood Feeding and Testing

*Cx. pipiens* egg rafts were originally collected in Pennsylvania in 2004 (courtesy of M. Hutchinson) and colonized at the arbovirus laboratory, Wadsworth Center (Albany, NY, USA). Mosquitoes were reared and maintained in 30.5 cm^3^ cages at 26 °C, 45%–65% relative humidity with a photoperiod of 16:8 (light:dark) hours and provided cotton pads with 10% sucrose *ad libitum*. A total of 300 5–7 day old female mosquitoes from the same generation were collected for each experimental group upon emergence, held in mesh top 3.8 L paper cartons, and deprived of sucrose for 24 h prior to blood feeding. All mosquitoes were fed on the same day on defibrinated chicken blood (Hema Resources, Aurora, Oregon, USA) with 2.5% sucrose together with either 1 mL EMEM (Earle’s Balanced Salt Solution, nonessential amino acids, 2 mM l-glutamine, 1 mM sodium pyruvate, and 1500 mg/L sodium bicarbonate [ATCC]) for the unexposed group, or 1 mL WNV strain NY1986 [23], amplified once on mosquito cell culture, and diluted in EMEM for the WNV exposed groups. Feeding of control and virus-challenged groups was carried out for 1 h using Hemotek membrane feeders (Discovery Workshops, Accrington, UK) with sausage casing heated to 37 °C. Mosquitoes were anesthetized using CO_2_ and only engorged individuals were maintained in pint cups with standard rearing conditions.

At 7 days post blood feeding mosquitoes were anesthetized with triethyamine (Sigma-Aldrich, St. Louis, MO, USA), separated, and individually surface sterilized in 1.8 mL tubes by using a single wash with 1 mL 70% EtOH followed by two washes with 1 mL phosphate buffer solution under sterile conditions. Following washes, individual mosquitoes were homogenized in 1 mL lysis buffer and DNA and RNA were extracted using an AllPrep DNA/RNA minikit (Qiagen, Valencia, CA, USA) according to the manufacturer’s protocol. The presence of WNV in the exposed mosquitoes was confirmed by reverse-transcription (RT)-PCR as previously described [24]. Mosquitoes were then separated into unexposed (UNEXP), WNV positive (WNV+) or WNV negative (WNV−) groups and DNA was used for bacterial 16S sequencing.

### 2.2. Bacterial Sequencing and Analysis

In order to assess the relationship between bacterial populations and WNV exposure and/or infection, deep-sequencing of bacterial 16S v3 and v4 hypervariable regions were completed for *Cx. pipiens* seven days post-feeding on blood meals, with or without virus, using the established MiSeq 16S pipeline (Illumina, San Diego, CA, USA). In short, 16S amplicons covering V3 and V4 hypervariable regions with overhang adapters were created via Taq PCR (New England Biolabs, Ipswich, MA, USA) and verified for size (459 bp), indices and Illumina sequencing adapters were attached using the Nextera XT Index kit (Illumina), and samples were normalized and pooled prior to sequencing at the Wadsworth Center Applied Genomics Core. Automated cluster generation and paired-end sequencing (250-bp reads) was performed on the Illumina MiSeq system. Analysis of 16S reads was completed using MiSeq Reporter, which utilizes the Greengenes 16S ribosomal RNA (rRNA) database for taxonomic assignments [25]. Additional microbiome analyses were completed at the Wadsworth Center Bioinfomatics Core using the open source pipeline Qiime v 1.9.0 [26]. Forward and reverse paired-end Illumina sequence data was combined using the join_paired_ends.py script. The command split_libraries_fastq.py was called to modify the fasta header identifiers to be Qiime compatible. Open reference OTU picking was done with pick_open_reference_otus.py using the default uclust method and the Greengenes OTU database. A mapping file was created to group the samples by experimental condition and used with subsequent analysis to process data. The command filter_samples_from_otu_table.py was used to create OTU tables (*i.e.*, biom files) with a minimum number of observations in each sample (20,000). Taxonomy plots were created with successive calls to summarize_otu_by_category.py, summarize_taxa.py and plot_taxa_summary.py. Alpha and beta diversity analysis and plots were created with core_diversity_analyses.py and specifying a sampling depth of 20,000.

GraphPad Prism v.5 was used for additional statistical analyses including chi-square tests used to compare proportion of OTUS among samples and groups, and Pearson’s correlation tests used to compare the relationships between proportions of individual genera.

### 2.3. Immune Transcript Quantification

Assays were performed according to standard protocol [27]. For each population (WNV+, WNV−, UNEXP), pools of 10 females from the original blood meal feeding groups were homogenized in 1 mL mosquito diluent (20% heat-inactivated fetal bovine serum (FBS) in Dulbecco’s phosphate-buffered saline (PBS) plus 50 μg/mL penicillin/streptomycin, 50 μg/mL gentamicin, and 2.5 μg/mL Fungizone) and briefly centrifuged. The supernatant was transferred to new tubes and total RNA was extracted with Trizol, quantified using a Nanodrop 2000 spectrophotometer (Thermo Scientific, Waltham, MA, USA) and subjected to reverse transcription using Superscript III (Invitrogen, Carlsbad, CA, USA) with random hexamers. Five microliters of complementary DNA (cDNA) was used for quantitative RT-PCR (qPCR). Real-time quantification was performed using the QuantiTect SYBR Green PCR Kit (Qiagen) and ABI Detection System ABI Prism 7500 (Life Technologies, Grand Island, NY, USA). Primer sequences have been previously published [28,29,30,31]. All qPCR reactions were performed in triplicate; to check for the specificity of the PCR reactions, melting curves were analyzed. The levels of expression in test samples were determined by normalizing results using the ribosomal rpl8 gene levels. The differential expression of REL1, HOP, DCR2, and TEP1 was calculated using the ΔΔCt method [32] following comparison to rpl8 expression. Results show the fold-difference of transcripts relative to the UNEXP group.

### 2.4. Total Microbial Load Determination

The amount of total bacterial DNA was determined in triplicate for individual mosquitoes using QuantiTect SYBR Green qPCR (Qiagen) with the same primers utilized for 16S sequencing (V3V4 hypervariable region). A standard curve was generated by extracting DH5-Alpha *Escherichia coli*, quantitating DNA utilizing a NanoDrop 2000 and calculating genome copies. A 10-fold serial dilution was created from 10^2^ to 10^8^ genome copies.

## 3. Results

### 3.1. Relationships between WNV Exposure and Microbial Signatures in *Cx. pipiens*

**Figure 1:**
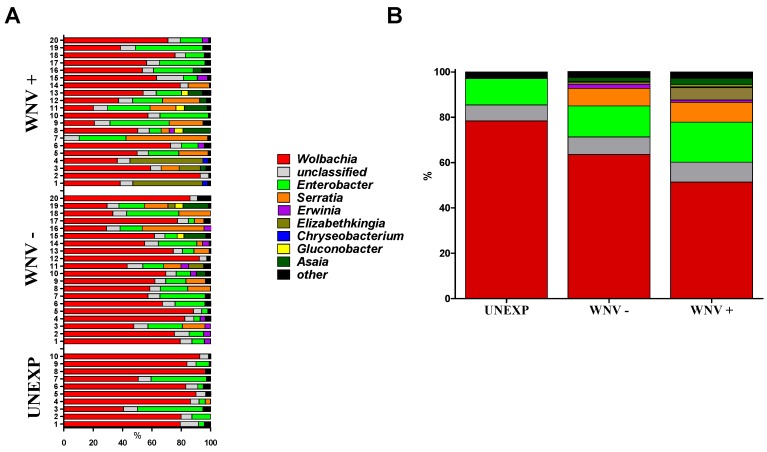
Proportions of bacterial genera identified in individual (**A**) or combined (**B**) *Cx. pipiens* at seven days post blood feeding. Mosquitoes are classified as unexposed (UNEXP, non-infectious blood meal), WNV negative (WNV−, WNV exposed but uninfected) or WNV positive (WNV+). Proportions were generated from 16S rRNA metagenomics workflow on MiSeq Reporter. “Other” refers to genera found at proportion below 3.5%.

**Figure 2:**
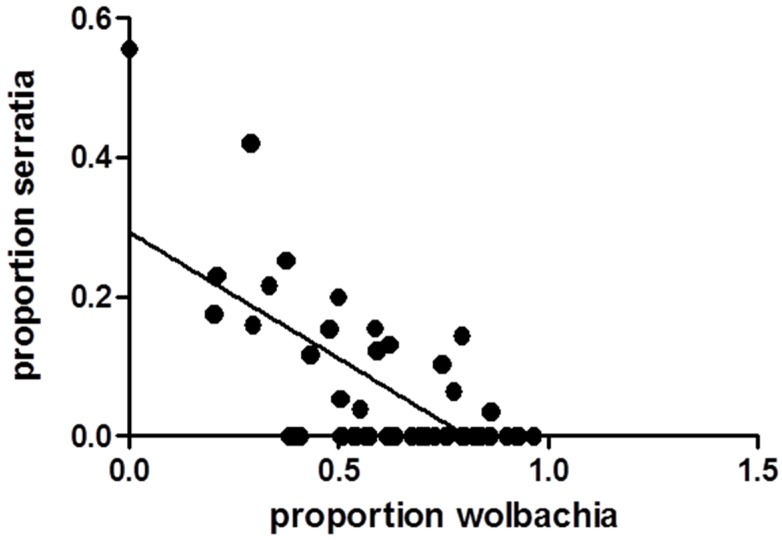
Relationship between the proportions of *Wolbachia* and *Serratia* in *Cx. pipiens* at seven days post blood feeding. Data points represent individual mosquitoes and the line represents the best-fit relationship resulting from linear regression analysis. A significant negative correlation was found (Pearson’s correlation, *r* = −0.687, *p* < 0.0001).

WNV titer of infectious bloodmeals was 7.3 log_10_ pfu/mL, a realistic natural dose [33]. 120 fully engorged *Cx. pipiens* were tested at day seven post-feeding for WNV RNA, of which 39 (32.5%) were WNV positive. Bacterial microbiome characterization was completed for a subset of these including 20 WNV+, 20 WNV− and 10 unexposed control (UNEXP) mosquitoes. A mean of 193,542 reads/sample were identified by Illumina metagenomics analyses as bacterial in origin and members of the Proteobacteria phylum represented over 96% all sequences (sequences available upon request). Substantial diversity was found within this phylum, with representatives from means of 61, 100, and 106 families, genera and species, respectively. Since the classification rate for the species level was less than 25% for all samples, genera were used for taxonomic assignments and diversity analyses. Results demonstrate substantial variability within and among groups in microbial compositions (Figure 1). Means of 85, 105, and 98 genera were identified in UNEXP, WNV− and WNV+ groups, respectively. Although experimental treatment was not associated with distinct phylogenetic signatures (Supplementary Figure S1), increased bacterial diversity was associated with WNV exposure and further elevated with WNV infection (Table 1; Supplementary Figure S2). When diversity is corrected for sampling (read depth) both Shannon entropy and Simpson’s diversity indices demonstrate a clear trend of increasing genus level diversity with UNEXP relative to WNV—and additionally with WNV—relative to WNV+ (Table 1). The most dominant genus was *Wolbachia* for all groups, comprising 61.7% of all reads, yet the mean percentage of *Wolbachia* in the UNEXP group (78.4) was significantly higher than the WNV− group (63.6; Chi-square, *p* < 0.001), which was further decreased with WNV infection (51.4; Figure 1). *Enterobacter*, the second most prevalent genus, was modestly higher with WNV exposure and infection, with percentages of 11.6, 13.6, and 17.6 in UNEXP, WNV−, and WNV+ groups, respectively. While none of the UNEXP samples had classifiable representatives outside of these two genera at proportions greater than 3.5%, 75% (30/40) of all WNV exposed (WNV+ and WNV−) mosquitoes had at least one other genus represented at this level. The most striking difference between the UNEXP and WNV exposed groups was in the genus *Serratia*. While just 0.36% of UNEXP sequences were identified as *Serratia*, 7.8% and 8.7% of the reads from WNV− and WNV+ were identified as *Serratia*, respectively, representing a greater than 20-fold increase in proportion in WNV exposed groups relative to the UNEXP group. There was a significant negative correlation between the proportions of *Wolbachia* and *Serratia* in the samples sequenced (Pearson’s correlation, *r* = −0.687, *p* < 0.0001; Figure 2). All reads belonging to these genera which were resolved on the species level were identified as *Wolbachia pipientis* and *Serratia entomophilia*.

**Figure 3:**
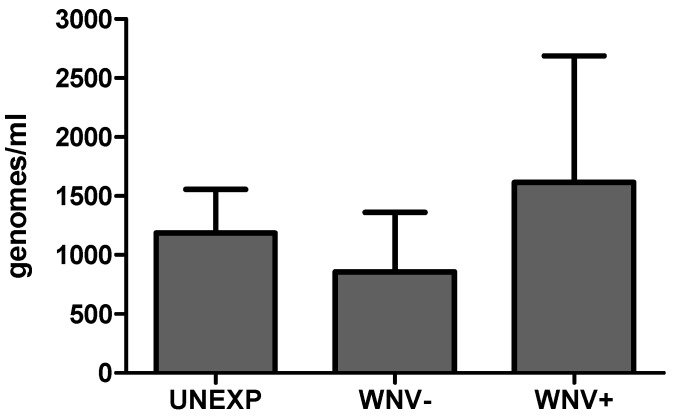
Total bacterial load in *Cx. pipiens* at seven days post blood feeding for unexposed (UNEXP), WNV−, and WNV+ mosquitoes. Bars represent means +/− SEM.

Quantitative PCR was used to assess if differences in diversity among groups correlate to generic differences in bacterial load. Results demonstrate significant variability in bacterial load among samples that did not consistently correlate to difference in bacterial diversity. Relative to the UNEXP group, mean bacterial load was modestly higher for the WNV+ group and modestly lower for the WNV− group, yet these differences were not significant (*t*-test, *p* > 0.05; Figure 3). Although it should be noted that the 16s copy number can be species-dependent to some degree, these results support the idea that neither WNV exposure nor infection status is associated with consistent variations in total bacteria. Interestingly, the individual mosquito with the highest bacterial load was the only sample in which no *Wolbachia* was identified and *Serratia* was the dominant genus.

**Table 1:** Bacterial diversity identified in *Cx. pipiens* seven days post blood feeding.

Group	Families/Sample	Genera/Sample	Species/Sample	1-D _1_	Sn _2_
UNEXP	54	85	83	0.37	0.052
WNV−	66	109	118	0.55	0.080
WNV+	60	98	105	0.68	0.102

_1_ Simpson’s diversity index (1-D), D = ∑n_i_(n_i_-1)/N(N-1), where n_i_=reads from individual genera and N = total number of reads. _2_ normalized Shannon entropy (S_n_) = ∑-_i_ P_i_ lnP_i_/ln N, where *p*_i_ = frequency of individual genera and N = total number of reads.

### 3.2. Mosquito Immune Gene Expression Following WNV Exposure and Infection

**Figure 4:**
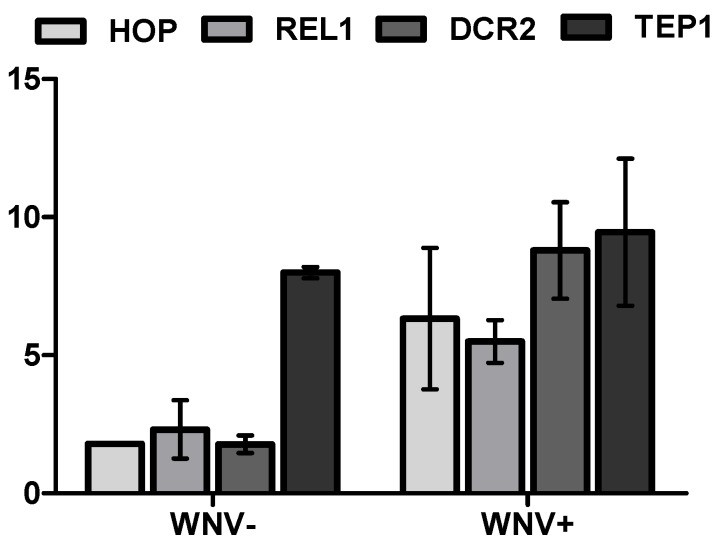
*Cx. pipiens* immune gene transcript levels at seven days post blood feeding for WNV+ and WNV− mosquitoes. Differences are expressed as mean fold change in qPCR CT (cycle threshold) +/− SD relative to unexposed mosquitoes and normalized by rpl8 transcript levels (ΔΔCT). Statistically significant differences (*t*-test, *p* < 0.001) were measured for all transcript levels for WNV exposed (− or +) relative to unexposed mosquitoes and WNV− relative to WNV+ mosquitoes, with the exception of TEP1, for which WNV− and WNV+ groups were statistically equivalent (*p* = 0.099).

In order to assess if WNV exposure and infection, as well as unique microbial signatures, were associated with variability in the regulation of innate immune pathways, transcript levels of markers of individual pathways including Jak-STAT (HOP), Toll (REL1), and RNAi (DCR2), as well as TEP1, were quantified from *Cx. pipiens* pools after experimental treatments identical to those used to attain individual microbial signatures. Values for ΔΔCT, which control for small differences in the housekeeping gene and are expressed as relative differences as compared to the UNEXP group, demonstrate that all four markers are up-regulated at seven days post WNV exposure (Figure 4, *t*-test, *p* < 0.01). An approximately two-fold increase was measured for HOP, REL1, and DCR2, while a much larger eight-fold difference in TEP1 expression was identified. More substantial increases in levels of expression of all transcripts were measured in the WNV+ group, with approximately 5–10-fold increases measured relative to the UNEXP group. With the exception of TEP1, all values were also higher in the WNV+ group relative to the WNV− group, and the largest difference measured between these groups was for DCR2 expression. Together these data demonstrate that there is a generic up-regulation of the mosquito immune response which coincides with increases in bacterial diversity and is sustained following WNV exposure, even in the absence of established infection.

## 4. Discussion

Interactions between resident microbial communities of mosquitoes and arboviruses can significantly alter mosquito immune and metabolic pathways and may influence many aspects of vectorial capacity including vector competence, blood feeding behavior, and longevity [3,22,34,35,36,37]. Although previous studies have largely focused on the capacity of *Wolbachia pipientis* to decrease fitness of DENV [38,39,40], WNV [41,42] and other arboviruses [43,44,45], there are likely numerous direct and indirect microbial interactions that could influence virus transmissibility [3]. Here, we add to the growing body of literature demonstrating such interactions, focusing on the effect of WNV exposure and infection on bacterial signatures in *Cx. pipiens*. Our results demonstrate that WNV infection increases the diversity of bacterial populations and is associated with up-regulation of classical invertebrate immune pathways. In addition, we offer the novel observation that WNV exposure without the establishment of infection results in lasting alterations to microbial and immune signatures.

While the effect of WNV on bacterial populations has not been previously assessed, a limited number of studies have characterized bacterial communities of *Culex* spp., focusing primarily on culturable bacteria [46,47,48]. Although colonized mosquitoes were used in the current study, as has been found in most studies with field mosquitoes, the dominant bacteria we identified are gram-negative Proteobacteria [37,47,48,49,50,51]. It is somewhat surprising that the levels of bacterial diversity in colonized mosquitoes are generally higher than those found in previous studies with field populations, yet this may be a result of methodology, as here we utilized next-generation sequencing technologies with significant depth (~200,000 reads/sample) and did not limit testing to the midgut or other individual tissues. Although *Wolbachia* was the dominant genus in the majority of mosquitoes, we also found substantial variability among individuals despite the homogeneity of rearing conditions. While competence is likely to always be stochastic to some degree, and mosquito genetics may also contribute, these unique microbial signatures could partially account for individual variability in WNV vector competence [22]. Although we did not identify any specific genera that were uniquely or consistently associated with WNV resistance in *Cx. pipiens*, results indicate that WNV exposure together with the previously documented effect of blood feeding [12] are likely to significantly disrupt populations, perhaps hindering the capacity to identify signatures of resistance at seven days post-feeding. The fact that the proportion of *Wolbachia* was significantly higher in WNV− mosquitoes relative to WNV+ mosquitoes stands in contrast to the idea that *Wolbachia* has the capacity to limit virus infection and spread, yet it has been demonstrated that this effect is strain-dependent [52,53]. In addition, it has been shown that *Wolbachia* conversely has the ability to enhance WNV infection in *Cx. tarsalis*, yet it should be noted that in these experiments *Wolbachia* was injected rather than stably-inherited and, therefore, likely resulted in altered tropism and biological consequences [54]. A comparison to unexposed mosquitoes suggests that lower *Wolbachia* levels are a result of WNV exposure, and more so infection, rather than a determinant of WNV competence. WNV could benefit from the capacity to directly inhibit *Wolbachia* [41], yet data suggest that differences in proportions of *Wolbachia* and other taxa more likely result from the indirect consequences of immune modulation [55,56,57] and/or resource competition associated with viral infection [58].

The association between WNV infection and increased bacterial diversity is consistent with a recent study with *Chikungunya virus* (CHIKV) and *Ae. albopictus*, which demonstrates an increase in *Enterobacter* together with a decrease in *Wolbachia* [51]. Similar to WNV results, this study additionally shows that the increased diversity is not necessarily associated with an overall increase in bacterial load but instead a shift in composition.

WNV infection was also associated with up-regulation of all major immune pathways evaluated here. RNAi is considered the primary invertebrate immune response associated with arbovirus infections [14]. Consistent with this is the increased expression of dicer2 (DCR2) in WNV+ mosquitoes relative to both UNEXP and WNV− mosquitoes. More surprising was the up-regulation of markers of other pathways historically associated with responding to bacteria and other microbes. These results are consistent with more recent studies demonstrating a broader role for these pathways, including in responding to viral infections [59]. Jak-STAT has been shown to restrict WNV as a result of secreted Vago in mosquito cells [60] and the Toll pathway has been implicated in control of DENV *Ae. aegypti* [61,62]. The Imd pathway has also been shown to play a role in defense against RNA viruses in *Drosophila* [63,64], yet is most often associated with activation by Gram-negative bacteria [65,66]. The broad reactivity of these pathways provides a plausible mechanism by which bacterial and viral populations might indirectly interact and result in reciprocal modifications. Indeed, the more mosquito microbiota are investigated the more it becomes clear that outcomes of individual infections likely need to be considered in the context of the holobiome, including not just arboviruses and bacterial populations, but also mosquito-specific viruses and fungi [67]. Further studies assessing microbial signatures and immune regulation together with WNV kinetics in *Cx. pipiens* will provide a clearer picture of the causal nature of such interactions.

Perhaps the most surprising result in the current study is the fact that WNV exposure alone is associated with lasting increases in bacterial diversity and immune gene expression in *Cx. pipiens*. Previous studies demonstrate that WNV resistance can be associated with decreased longevity of *Cx. pipiens*, suggesting initial infection may be established and quickly quelled, but at a fitness cost [16,68]. One plausible explanation for this cost offered by the current data is that the lasting immune activation may come at a metabolic price, as has been seen with studies of pathogen resistance in *Drosophila* [69]. Alternatively, costs of both resistance and infection could be “multiple fronts costs” in which the immune response to the invading virus decreases the capacity to keep particular bacteria below pathogenic levels [70].

Although there was substantial variability among individual mosquitoes, the most consistent and sizable increase identified in WNV exposed mosquitoes was with the proportion of *Serratia*. Similar to *Wolbachia*, *Serratia* spp. have been shown to be passed both vertically and transtadially by mosquitoes [71] and *S. odorifera* have been demonstrated to enhance susceptibility of *Ae. aegypti* to both CHIKV and DENV with co-feeding experiments [71,72]. *S. marcescans* has the capacity to modulate *Plasmodium* infection and be pathogenic to mosquitoes [73,74,75], as well as having other antimicrobial properties [76,77]. A significant negative correlation between *Wolbachia* and *Serratia* was observed. This is consistent with there being a competitive interaction between these microbes, as has been observed with *Wolbachia* and other co-infecting endosymbionts [78,79], as well as with other maternally inherited bacteria [80,81]. A recent study demonstrated inhibition of *Wolbachia* by *Asaia* bacteria in *Anopheles* mosquitoes [82], a genus which was also identified in a number of WNV exposed mosquitoes in the current study. *Serratia* sequences for which species-level classification was possible in the current study were identified as *S. entomophilia*. Although this species has not been previously associated with mosquitoes, it is documented to be pathogenic in coleopteran larvae [83] and has been commercially used as an insecticide [84]. While the relationship between bacterial composition and vector competence is frequently discussed, the possibility that interactions among bacteria and/or between bacteria and viruses could alter other aspects of vectorial capacity, including vector longevity, has generally been overlooked. Further investigation of these interactions using field populations will help elucidate the likelihood that such relationships could ultimately be exploited in novel control strategies.

## Supplementary Files

Supplementary File 1
