# Supplementary material for: Exposure to West Nile Virus Increases Bacterial Diversity and Immune Gene Expression in Culex pipiens
Source: Viruses. 2015 Oct 27;7(10):5619–31. doi: 10.3390/v7102886 (PMC4632394; doi:10.3390/v7102886)
Supplement: Supplementary File 1 [file viruses-07-02886-s001.pdf]

viruses

MDPI

Supplementary Materials

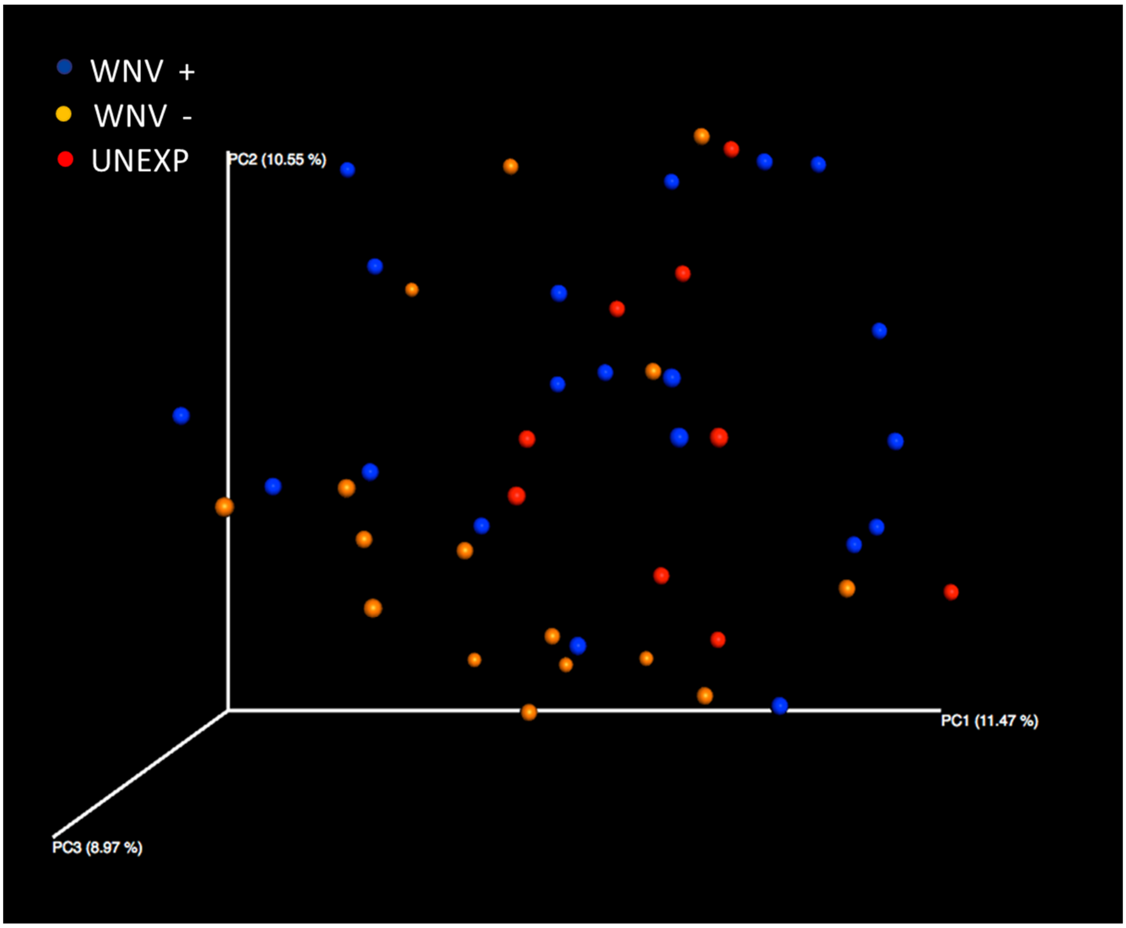

Supplementary Figure 1. PCoA plots for *Cx. pipiens* microbiome at 7 days post blood feeding for unexposed (UNEXP), WNV negative (−) and WNV positive (+) mosquitoes. Plots were generated by Qiime v1.09.

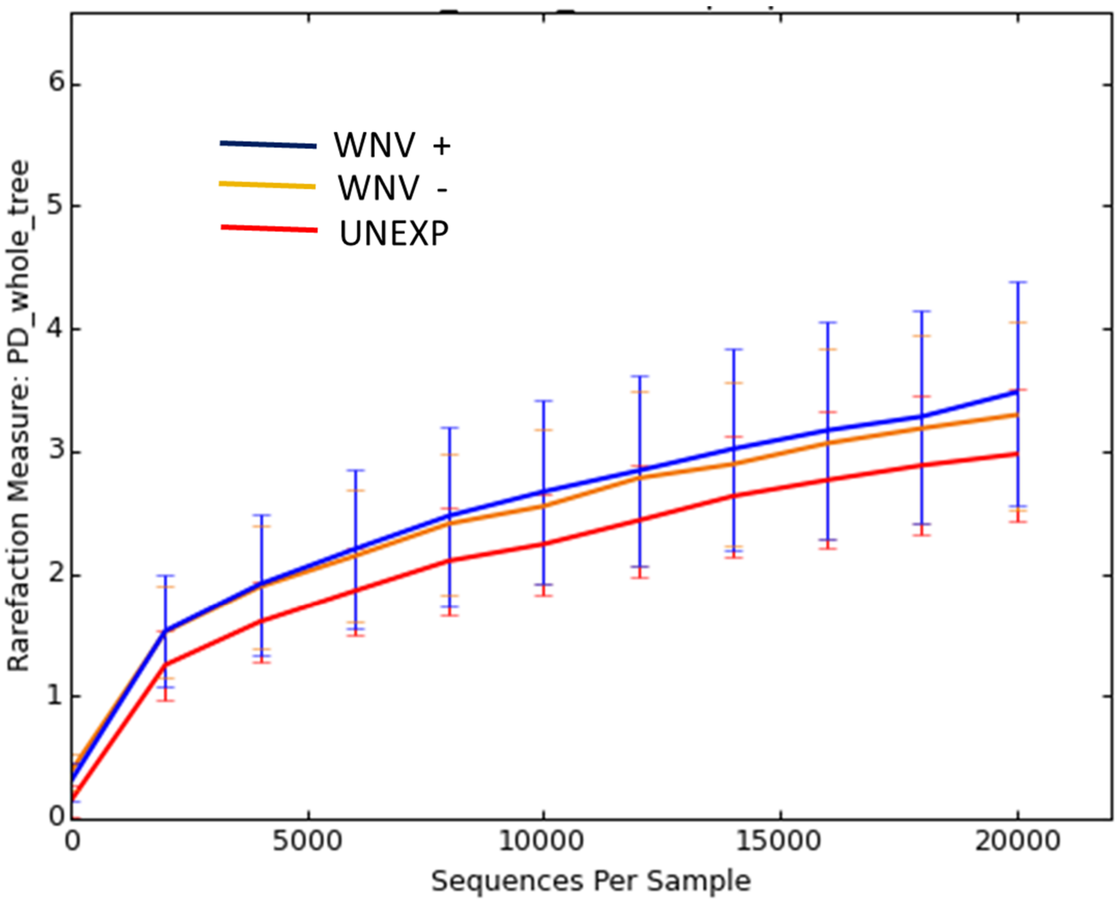

Supplementary Figure 2. Alpha diversity *vs.* sequence depth for *Cx. pipiens* microbiome at 7 days post blood feeding for unexposed (UNEXP), WNV negative (−) and WNV positive (+) mosquitoes. Plots were generated by Qiime v1.09.

© 2015 by the authors; licensee MDPI, Basel, Switzerland. This article is an open access article distributed under the terms and conditions of the Creative Commons by Attribution (CC-BY) license (http://creativecommons.org/licenses/by/4.0/).
